# Translation and Validation of the Spiritual Care Intervention-Provision Scale in the Greek Language

**DOI:** 10.1155/2020/8568043

**Published:** 2020-10-07

**Authors:** Evangelos C. Fradelos, Ioanna V. Papathanasiou, Foteini Tzavella

**Affiliations:** ^1^Nursing Department, University of Peloponnese, Tripoli, Greece; ^2^Nursing Department, University of Thessaly, Larissa, Greece

## Abstract

**Introduction:**

Spiritual care is defined as activities and interventions that promote spiritual health and the spiritual dimension of quality of life. Empirical data indicate the importance that spiritual care provision has on nursing practice. The spiritual care intervention-provision (SCIP) scale was developed to assess the frequency of spiritual care intervention implemented by nurses. Currently, there are no validated scales for assessing spiritual care in the Greek language.

**Objective:**

To adapt and validate the spiritual care intervention-provision scale in the Greek language.

**Methods:**

A descriptive cross-sectional study was employed, in which 275 nurses working in two public hospitals participated. The SCIP scale underwent the process of cross-cultural adaptation and was evaluated by assessing its reliability and validity.

**Results:**

The process resulted in a valid Greek version of the SCIPS, the internal consistency (Cronbach's *α* 0.887), reliability testing-retesting (*r* = 0.997, *p* < 0.001, and *t* = 0.387, *p* > 0.05), construct, and convergent validities were evaluated.

**Conclusion:**

The Greek version of the spiritual care intervention-provision scale is a validated scale that can be used to examine spiritual care provision in Greek health services.

## 1. Introduction

Although research interest in spirituality in the field of health sciences has been growing over the recent years, in nursing science and in everyday nursing clinical practice, spirituality has always been an integral part, not only since it was defined and organized as a science [1], but previously as well [2, 3]. It is worth noting that, for centuries, healthcare was delivered in temples, monasteries, and places of worship in general [4], thus, consolidating and perpetuating the already strong relationship between health, nursing, and spirituality. Over the years, healthcare delivery may have shifted from religious structures to a more organized system; however, in the late 1960s, emphasis was given to spirituality and spiritual care provided by nursing professionals. For example, the Christian nurses association launched a series of certified courses and seminars on spiritual care provision in the context of healthcare, which demonstrated the importance that nurses give to patients' spiritual needs [5].

There are many definitions of spiritual care. According to Dhamani, Paul, and Olson, spiritual care is including “activities and interventions that promote spiritual health and the spiritual dimension of quality of life [6].” However, according to another definition, spiritual care is a type of care that promotes spiritual health and relieves stress, which affects the spiritual dimension of an individual or a community. Therefore, spiritual nursing care supports spiritual health and aims to maintain the balance between a person's psychosocial and spiritual dimensions while providing a sense of wellbeing and integrity [7]. According to a comprehensive recent definition provided by Ramezani et al. [8], spiritual care is a subjective and dynamic concept of nursing care, integrating all the other aspects. Furthermore, they argue that spiritual care includes seven defining attributes such as healing presence, the therapeutic use of self, intuitive sense, exploration of the spiritual perspective, patient-centredness, meaning-centred therapeutic intervention, and the creation of a spiritually nurturing environment [8]. Nevertheless, according to empirical data, by providing spiritual care to their patients, nurses preserve patients' humanity, dignity, and integrity. They help patients give meaning to their lives and illnesses [9, 10]. Furthermore, spiritual care intervention, such as psychotherapy and meditation, can enhance patients' quality of life, decrease stress, and provide relief from depressive and anxiety symptoms [11, 12].

Many educational and professional nursing organizations include spirituality and spiritual care in the various declarations and guidelines they issue regarding proper nursing practice. The American Association of Nursing Colleges has included training on spirituality and spiritual care in the core courses of nursing, while the American Nursing Association delivers extensive talks on these issues in its publications, such as Nursing Practice Standards, the Social Policy Statement for Nurses, and the Code of Conduct for Nurses [1]. Internationally, both spirituality and spiritual care have been recognised by international health organizations, such as the International Nursing Council (ICN) and the World Health Organization (WHO), for their beneficial effects on the health and wellbeing of the individual [4, 13]. Modern nursing literature seeks to clarify the term spirituality through qualitative studies in various clinical practice contexts and also to highlight the effectiveness and quality of nursing care provided by the detection of patients' individual spiritual needs [14].

Researches worldwide have reported the health benefits of incorporating spirituality in healthcare and providing spiritual care in the hospital setting. According to a cross-sectional quantitative study in Iran, in which 271 ICU patients participated, patients' spirituality and religiosity are negatively related to the development of anxiety, depression, and posttraumatic stress disorder [15]. In addition, the study argued that patients'religiosity and spirituality can influence coping abilities, medical decisions, and treatment compliance, even making it possible to affect medical outcomes. Moreover, they argued that it is crucial to meet patients' spiritual needs because failing to do so increases healthcare costs [15]. Within the context of another quantitative study addressing the issue of spiritual care among HIV + populations, its benefits on mental and physical health were highlighted. More specifically, Farzanegan et al. [16] observed that providing HIV+patients with spiritual care and enhancing patients' religiosity and spirituality can have positive effects on patients' social support, mental health outcomes, and adherence to treatment. In addition, it was observed that patients with higher scores in religiosity and spirituality had better immune faction and improved CD-4 count [16]. Furthermore, the incorporation of spirituality and religiosity within healthcare delivery is an emerging issue from the patients' perspective as well. A multicentre observational study among 326 hospitalised patients in Springfield USA, noted that patients had religious and spiritual concerns during their hospitalisation, and almost half of them were using religious resources, such as chaplain visits, during their hospitalisation [17].

Although the issue of spirituality and spiritual care in nursing practice has been urged from empirical data and by professional organizations, patients' spiritual needs are often neglected. According to a study by Stranahan [18], which aimed to investigate the attitudes of nurses toward spiritual care, 57% of participants rarely or do not provide any kind of spiritual care. In addition, this attitude has been found to be related to inadequate training in providing such care. Thus, it was concluded that the undergraduate and graduate education provided is insufficient to prepare students for such interventions. Finally, it proposed the development of special courses in spiritual care to prepare students for its provision. Furthermore, a qualitative study conducted by Tanyi [19], in which various health professionals participated, found that the reported lack of specific training on this subject was one of the main reported barriers to care. They also proposed the development of special educational programs, interventions based on data, and dissemination of this information in the professional field, in order to raise the awareness of health professionals.

Considering the importance of spiritual care provision reported in various studies [20], many studies in Greece support the importance of spirituality in patients' healthcare, its association with health outcomes [13, 21, 22], and the lack of a valid instrument to measure nurses' spiritual care provision and intervention. Hence, the present study aims to assess the psychometric properties and validate the Greek version of the SCIP scale. The SCIP scale is a brief, easy to administer the instrument that assesses both existential and religious care. After being validated, the scale will be used to understand the nurses' attitudes toward spiritual care and examine factors associated with spiritual care provision.

## 2. Methods

### 2.1. Study Design and Participants

A descriptive, cross-sectional study for the assessment of psychometric properties and validation of the SCIP scale was conducted. The required sample size was calculated by fulfilling the following requirements. The recommended 1 : 10 ratio of the number of items to the number of participants, as suggested by Terwee et al. [23], was selected. Considering a sample size of 300 participants, to be fair, a contact factor analysis was included in the validation process [24]. Nurses and nurse assistants willing to participate working in two hospitals, located in Athens, the capital of Greece, were selected. The duration of the study was from October to November of 2019. The questionnaires were distributed randomly to 350 nurses in two hospitals. 297 participants filled out the survey (84% response rate), and a total of 275 questionnaires were completed (78% completion rate). From those, a purposeful sample of 50 participants completed the questionnaire for a second time after three weeks in order to assess reliability. The inclusion criteria were required one to be a nurse or a nurse's assistant, currently working in the selected two hospitals, with a minimum of one-year working experience.

### 2.2. Ethical Considerations

This study was approved by the ethics committees of both hospitals as well as the ethical committee of the Faculty of Health Science of the University of Peloponnese. The questionnaires were anonymous and self-administered. Nurses meeting the inclusion criteria were verbally requested to participate in the study. Each participant was informed about the purpose of the study and the right to take part, refuse, or withdraw at any time.

### 2.3. Instruments

The instrument included a sheet containing the samples' sociodemographic and work-related characteristics.

#### 2.3.1. Spiritual Care Intervention-Provision Scale (SCIP Scale)

The SCIP scale is a 17-item self-administered scale, developed by Musa and Pevalin [25], and was used to measure the frequency of nurses providing spiritual care to their patients. The initial version was culture and religion specific, so minor modifications had to be made during the cultural adaptation process. It is a two-factor scale, one factor for assessing existential spiritual care intervention-provision, consisting of nine items (ESCIP) and one for religious, spiritual care intervention-provision (RSCIP), consisting of eight items. Answers are given on a four-point Likert scale, ranging from “never” (1), “rarely” (2), “sometimes” (3), to “always” (4). The scale shows an overall SCIP score that is computed by adding the responses to all 17 items, with the total score ranging from 17 to 68, with higher scores indicating a greater provision of spiritual care interventions. In addition, two more scores can be computed—the ESCIP, ranging from 9 to 36, and the RSCIP, with a score ranging from 8 to 32. During the development procedure, the scale exhibited acceptable reliability, with values of Cronbach's *α* ranging from 0.77 to 

In order to assess convergent validity, two instruments were selected: the caring behaviors inventory that assesses four dimensions of caring and the functional assessment of chronic illness therapy-spiritual wellbeing scale-12 nonillness to assess nurses' spirituality [26, 27].

The caring behaviors inventory (CBI) was used to measure nurses' caring behaviors. CBI is a 24 items questionnaire that assesses four dimensions of nurse caring behaviors, namely, (1) assurance of human presence, (2) professional knowledge and skills, (3) patient respectfulness, and (4) positive connectedness. Answers are given on a six-point Likert scale, ranging from 1 = never to 6 = always. A higher score in a domain means that the nurse is exhibiting the corresponding caring behavior. In this study, the Greek version was used as it was adopted by Papastavrou et al. [28].

The functional assessment of chronic illness therapy-spiritual wellbeing scale-12 nonillness (FACIT-Sp-12) was used to assess nurses' spirituality. This scale is used to measure three dimensions of spirituality, namely meaning, peace, and faith, and it provides a total score. This scale evaluates over a seven-day period; it is a twelve-item questionnaire and responses are given on a four-point Likert scale, ranging from 0 “not at all” to 4 “very much.” Higher scores in the total scale and in the three domains indicate higher spirituality. The Greek version of FACIT-Sp-12 used was validated by Fradelos et al. and has been used both for healthy individuals and those suffering from chronic conditions [30, 31].

### 2.4. Translation and Cultural Adaptation Procedure

The translation and cultural adaptation were carried out according to the WHO's translation, cross-cultural, and linguistic validation methodology [32]. Two bilingual translators produced two separate versions of the SCIP scale in Greek. The two versions were reconciled by a third individual into a final version in Greek. During the forward translation, modifications were made on three items of the SCIP. Quran is not a regular religious book for Greek nurses, instead the phrase “holy texts” or “books” was used to replace the Quran. In the next step, the Greek version was translated back into the English by two bilingual native English speakers, and it was checked for inconsistencies. Next, the final Greek version (Appendix) was piloted on a purposeful sample of 10 Greek registered nurses in one of the hospitals where the study was conducted. All nurses found the scale easy to understand and suitable. Therefore, no further changes were made to this version.

### 2.5. Statistical Analysis

The SPSS v. 25 used to perform the statistical analysis of the study. Quantitative variables are presented as means (±standard deviation) and qualitative variables as absolute and relative frequencies. For internal consistency, the scale was assessed with a Cronbach's *α* coefficient, and the reliability of the SCIPS was assessed using the test-retest method (*n* = 50). Exploratory factor analysis (EFA) with Varimax rotation of axis was applied to determine the factorial structure of the SCIPS (*n* = 275). Varimax rotation is a very common rotation and it was chosen because it simplifies the loadings of items by removing the middle ground and, more specifically, identifying the factor upon which data load. Convergent validity was assessed by the degree of correlation of SCIPS with CBI 24 and FACIT-Sp-12, as it was assumed that increased SCIPS will be positively correlated with nurses' spiritual wellbeing and their caring behaviors [26, 27]. Reported *r* values between 0 and 0.3 (0 and −0.3) indicate a weak linear relationship, values between 0.3 and 0.7 (−0.3 and −0.7) indicate a moderate, and values between 0.7 and 1.0 (−0.7 and −1.0) indicate a strong linear relationship.

## 3. Results

A total of 350 nurses were approached during the data collection period, of which 297 provided data for the study (84% response rate), and only 275 of them completely filled the questionnaire. From a total of 275 nurses, 238 were women (86.5%) and the mean of age was 43.8 ± 8.7, 64.4% were married, and the majority of them (76.3%) had completed their university studies. Their work experience was found to be 19.9 ± 9.7 years, and most of the participants were working in medical departments (*n* = 124, 45.1%). There were 179 (65.1%) staff nurses and 71 (25.8%) nurse assistants, while 23 (8.4%) of them were head of departments and 2 (0.7%) were sector supervisors.

### 3.1. Scale Reliability

The reliability of the SCIPS scale was tested for characteristics of stability and internal consistency. The test-retest method was used to examine scales stability and Cronbach *α* coefficient to examine internal consistency. From a total of 275 nurses, 50 of them completed the scale for a second time (retest) after a three-week period. A sufficient period of time had passed so that there was no remembrance of the previous answers. For testing the repeatability of measurements between the test and retest, Pearson's correlation coefficient was estimated, and a paired *t*-test was employed for the difference between the two administrations of the scale. Strong correlations were seen between the two administrations of the scale, between the total score of the scales (*r* = 0.997 and *p* < 0.001) and partial sums of the subscales (*r* = 0.989 and *p* < 0.001; ESCIPS *r* = 0.993 and *p* < 0.001 for RSCIPS). In addition to *t*-values in the paired *t*-test between the two administrations, the total score of the scales and the partial sums of the subscales were not statistically significant. Thus, we can say there were no statistically significant differences between the two administrations so the questionnaire has good test-retest reliability, meeting the characteristic of stability. The results of the test-retest reliability are shown in Table 1.

### 3.2. Internal Consistency

For testing the internal consistency of the SCIPS, the Cronbach's *α* coefficient was used. The internal reliability coefficient for the total score of the SCIPS scale was 0.887, demonstrating good internal consistency. The internal consistency coefficient (Cronbach's *α*) for each factor of the Greek version of the SCIPS was 0.854 for ESCIPS and 0.850 for RSCIPS. The scale and the subscales came to >0.70, supporting that the scale has satisfactory internal consistency [33]. Moreover, values of Cronbach's *α* in all subscales, in case one item was deleted from the subscale, were checked. The audit showed that no substantial increase in the Cronbach's *α* will happen if an item is deleted from the subscales. Thus, we can say that all the questions were important for internal coherence with the other. Finally, the mean scores for the total scale were 47.20 ± 9.13, 29.40 ± 4.76 for ESCIPS, and 17.80 ± 5.68 for the RSCIPS. The results are presented in Table 2.

### 3.3. Construct Validity of the Greek Version of SCIPS

Exploratory factor analysis was applied to explore the construct validity of the scale. For extracting the factors, principal component analysis (PCA) with Varimax rotation axes method was applied. The high value of the KMO index (KMO = 0.876) and Bartlett's Test of Sphericity (*X*^2^(136)=2138.999, *p*=0.001) values support the sample adequacy. The factor analysis resulted in two factors, with eigenvalue>1 (Kaiser criterion) that interpreted 52.132% of the total variance. All item loadings in the factors had values >0.40, which is the marginal acceptance point. Moreover, none of the items had above 0.45 loading in more than one factor. Thus, all items had significant loadings and could be included as factors. The first factor fully corresponding to the ESCIPS factor of the initial scale had an eigenvalue of 6.192 and 36.426% total variance (Q. 1, 2, 10, 12, 13, 14, 15, 16, 17). The second factor fully corresponds to the RSCIPS factor of the initial scale with eigenvalue 2.670 and 15.706% total variance (Q. 3, 4, 5, 6, 7, 8, 9, 11). Detailed results are presented in Table 3.

### 3.4. Convergent Construct Validity

Convergent construct validity was examined through hypothesis testing. It was assumed that the SCIP total and its subscale will positively correlate to the nurse's spirituality and caring behaviors—the SCIPS was found to correlate as expected. The SCIPS total, ESCIPS, and RSCIPS exhibited some weak and many moderate but yet statistically significant correlations with the nurse's total spirituality, meaning, peace, and faith as well as with assurance of human presence, professional knowledge and skills, patient respectfulness, and positive connectedness. Detailed results are presented in Table 4.

## 4. Discussion

The aim of the present study is the cross-cultural adaptation and validation of the SCIP scale in Greek. For the validation of the scale, specific psychometric tests were performed to assess the reliability of the questionnaire and validity, while at the same time, the dimensionality of the scale was tested as well (factor analysis). Reliability was assessed via a test-retest method and the Cronbach's *α* index. In addition, the convergent construct validity was assessed. From the mentioned statistical test, we conclude that the Greek version of the scale is a valid and reliable tool to assess nurses' spiritual care provisions.

Spiritual care is an important element in holistic nursing care. It is also recognised as a significant factor in the psychosocial needs of people, mainly for individuals dealing with life-threatening and end-stage diseases. Religious and spiritual beliefs can have a positive effect on life by acting as a source of inspiration. Spiritual care can also reflect the multifaceted expressions of spirituality that healthcare professionals are called to address for their patients and their families. Specialised spiritual care often includes understanding and assistance of specific theological beliefs, sometimes being performed ideally by individuals in the special education field [34]. The integration and assessment of a patient's spirituality during healthcare delivery can play a vital role. It can be a dynamic cause in the patient's coping with the disease and give meaning and propose to their life and illness. Spiritual and religious beliefs are often associated with various health related decisions, and understanding the patient's spirituality is integral to the whole system of patient care [35]. The mean values of SCIPS, RSCIPS, and ESCIPS are 47.20 ± 9.13, 17.80 ± 5.68, and 29.40 ± 4.76, respectively. These values are the theoretical mean values; thus, we can argue that Greek nurses often provide spiritual care for their patients and mostly existential spiritual care. In the scale development study by Musa and Pevalin [25], as well as in other studies conducted in similar cultural environments [36, 37], nurses scored higher values in the SCIP scale in comparison with our results. This finding can be culturally explained and by the perceptions that nurses have, which is providing spiritual care. Although the ESCIPS value is satisfactory, it is worth mentioning that in all these studies, nurses are reporting a lower RSCIP subscale score. Our results agree with these abovementioned studies. Maybe this difference in scores can be explained by the fact that nurses expect some of these needs to be addressed by experts, such as priests and religious leaders [36, 38].

In terms of the reliability of the SCIPS scale, it was assessed by the characteristics of stability and internal consistency. Both the test-retest procedure and the values of the Cronbach's *α* for the total scale and the two subscales were found to be 0.70, thus, supporting the scales' reliability. The Cronbach's*α* value was 0.887, 0.854, and 0.850 for the total scale, ESCIPS and RSCIPS, respectively. Those values are similar to the values reported in the original survey by Musa and Pevalin, during the scales' development [25], except the RSCIPS' Cronbach's *α* that, in the present study, is higher than the original survey. This fact supports greater internal consistency of the validated instrument. Regarding the dimensionality of the instrument, our results are in agreement with the original version of the scale, and the exploratory factor analyses resulted in a two-factor solution that corresponds to the original scales' structures [25]. Although this specific scale has not been validated in many languages, there are much research and empirical data in the field of spirituality and spiritual care that support the existence of these two components of ESCIP and RSCIP [39–41]. The Greek version of SCIPS in total, and individual subscales, was found to be positively correlated with the nurses' spirituality and caring behaviors. These results reinforce and validate previous studies that were suggesting nurses with higher levels of spirituality are more likely to understand patients' spiritual needs and provide spiritual care [39, 42]. Moreover, our results on the positive relationship between spiritual care and nurses' caring behaviors agree with previous studies. According to a cohort study conducted among nursing students by Hoover, the increased level of spiritual awareness can result in a more comprehensive understanding of caring from a theoretical perspective, a more holistic approach to care and enhance caring skills [43]. Furthermore, according to a more recent cross-sectional study that explored the relationship of nurses' religiosity, spirituality, and nursing care among Taiwanese nurses, it showed that nurses' spiritual health can positively influence their attitudes toward spiritual care, professional commitment, and caring [43] The mentioned studies indicate the need to address patients' spiritual needs in enhancing nurses' caring. However, more research is needed to validate such claims.

Nonetheless, some limitations to this study should be noted. Although the sample was considerably fair, the SCIPS was tested on a convenience sample of Greek nurses; therefore, the generalisation of the findings to the total population of Greek nurses cannot be done. In addition, we only perform exploratory and confirmatory factor analysis. These two limitations should direct future researches that aim to explore spiritual care in Greek nurses. This study also has two major strengths worth mentioning; first, the sample size for the validation process was fair and second, the issue of spiritual care provision has been addressed for the first time in quantitative research in Greece.

## 5. Conclusions

The Greek translation of the SCIP scale is a brief, easy to administer assessment instrument that assesses nurses' spiritual care provision. It is demonstrated to be a valid and reliable instrument for assessing the frequency of nurses providing spiritual care for their patients, as it is supported by excellent psychometric properties [29].

## Figures and Tables

**Table 1:** Test-retest reliability of spiritual care intervention-provision scale (3-week period between test-retest, *n* = 50).

SCIPS	Test (Α) Mean ± st. Dev.	Retest (Β) Mean ± st. Dev.	Pearson's *r* Correlation between (Α) and (Β)	Paired *t*-test (*t*) difference (Α) and (Β)	Intraclass correlation coefficient (ICC)
ESCIPS	29.08 ± 4.98	28.88 ± 4.74	0.989^*∗∗*^	0.871^*∗*^	0.978^*∗∗*^
RSCIPS	16.1 ± 6.29	17.48 ± 6.95	0.993^*∗∗*^	0.745^*∗*^	0.988^*∗∗*^
SCIPS	47.42 ± 11.23	47.36 ± 10.63	0.997^*∗∗*^	0.387^*∗*^	0.978^*∗∗*^

^*∗*^ *p* > 0.05^*∗∗*^*p* < 0.001

**Table 2:** Internal consistency of the SCIPS (*n* = 275).

	Range	Minimum	Maximum	Mean ± SD	Cronb.a
SCIPS total	49.00	19.00	68.00	47.20 ± 9.13	0.887
ESCIPS	26.00	10.00	36.00	29.40 ± 4.76	0.854
RSCIPS	24.00	8.00	32.00	17.80 ± 5.68	0.850

**Table 3:** Items loadings in factor analysis of the spiritual care intervention-provision Scale (*n* = 275).

SCIPS scale	Factor 1“ESCIPS ”	Factor 2“RSCIPS”
Item 1	0.654	0.180
Item 2	0.660	0.320
Item 3	0.280	0.655
Item 4	0.240	0.689
Item 5	0.120	0.713
Item 6	0.150	0.786
Item 7	0.320	0.752
Item 8	0.230	0.725
Item 9	0.250	0.627
Item 10	0.670	0.330
Item 11	0.250	0.640
Item 12	0.613	0.360
Item 13	0.639	0.150
Item 14	0.882	0.090
Item 15	0.850	0.070
Item 16	0.765	0.230
Item 17	0.796	0.300
Eigenvalue	6.192	2.670
% Variance	36.426	15.706

ΚΜΟ = 0.876. Bartlett's test: *χ*2 (136) = 2138.999 *p* < 0.001 Principal component analysis with the varimax method.

**Table 4:** Correlations between the scores of the SCIPS and the FACIT-SP12 and CBI subscales.

	Functional assessment of chronic illness therapy-spiritual wellbeing scale-12 nonillness				Caring behaviors inventory			
	Total spirituality	Meaning	Peace	Faith	Assurance of human presence	Professional knowledge and skills	Patient respectfulness	Positive connectedness
SCIPS total	0.266^*∗∗∗*^	0.234^*∗∗∗*^	0.155^*∗∗*^	0.222^*∗∗∗*^	0.362^*∗∗∗*^	0.239^*∗∗∗*^	0.396^*∗∗∗*^	0.428^*∗∗∗*^
ESCIPS	0.238^*∗∗∗*^	0.274^*∗∗∗*^	0.096	0.201^*∗∗∗*^	0.393^*∗∗∗*^	0.297^*∗∗∗*^	0.480^*∗∗∗*^	0.474^*∗∗∗*^
RSCIPS	0.227^*∗∗∗*^	0.148^*∗*^	0.169^*∗∗*^	0.189^*∗∗*^	0.252^*∗∗∗*^	0.135^*∗*^	0.234^*∗∗∗*^	0.291^*∗∗∗*^

^*∗*^ *p* < 0.05^*∗∗*^*p* < 0.01^*∗∗∗*^*p* < 0.001

## Data Availability

The data used to support the findings of this study are available upon request.

## References

[1] Bennett V., Thompson M. (2015). Teaching spirituality to student nurses. *Journal of Nursing Education and Practice*.

[2] Connell Meehan T. (2012). Spirituality and spiritual care from a Careful Nursing perspective. *Journal of Nursing Management*.

[3] Burkhart L., Hogan N. (2008). An experiential theory of spiritual care in nursing practice. *Qualitative Health Research*.

[4] Zyga S. (2015). Assessing patients spirituality: a new age holistic approach or a forgotten nursing practice?. *Health Science Journal*.

[5] Shelly J. A., Fish S. (1988). *Spiritual Care: The Nurse’s Role*.

[6] Dhamani K. A., Paul P., Olson J. K. (2011). Tanzanian nurses understanding and practice of spiritual care. *ISRN Nursing*.

[7] McEwen M. (2005). Spiritual nursing care. *Holistic Nursing Practice*.

[8] Ramezani M., Ahmadi F., Mohammadi E., Kazemnejad A. (2014). Spiritual care in nursing: a concept analysis. *International Nursing Review*.

[9] Watson J. (2012). *Human Caring Science: A Theory of Nursing*.

[10] Timmins F., Caldeira S. (2017). Understanding spirituality and spiritual care in nursing. *Nursing Standard*.

[11] Gonçalves J. P. B., Lucchetti G., Menezes P. R., Vallada H. (2015). Religious and spiritual interventions in mental health care: a systematic review and meta-analysis of randomized controlled clinical trials. *Psychological Medicine*.

[12] Vallada J., Lin Y., Yan J., Wu Y., Hu R. (2018). The effects of spiritual care on quality of life and spiritual well-being among patients with terminal illness: a systematic review. *Palliative Medicine*.

[13] Fradelos E., Tzavella F., Koukia E. (2015). Integrating chronic kidney disease patient’s spirituality in their care: health benefits and research perspectives. *Materia Socio Medica*.

[14] Pullen L., McGuire S., Farmer L., Dodd D. (2015). The relevance of spirituality to nursing practice and education. *Mental Health Practice*.

[15] Bashar F. R., Vahedian-Azimi A., Vahedian-Azimi A. (2018). Spiritual health and outcomes in muslim ICU patients: a nationwide cross-sectional study. *Journal of Religion And Health*.

[16] Farzanegan V., Hung L. C., Abbasgholizadeh R., Terrell Hamilton F., Essien E. J., Nwulia E. (2017). Spiritual care may impact mental health and medication adherence in HIV+ populations. *HIV/AIDS—Research and Palliative Care*.

[17] Ellis M. R., Thomlinson P., Gemmill C., Harris W. (2012). The spiritual needs and resources of hospitalized primary care patients. *Journal of Religion and Health*.

[18] Stranahan S. (2001). Spiritual perception, attitudes about spiritual care, and spiritual care practices of nurse practitioners. *Western Journal of Nursing Research*.

[19] Tanyi R. A. (2002). Towards clarification of the meaning of spirituality. *Journal of Advanced Nursing*.

[20] Babamohamadi H., Ahmadpanah M.-S., Ghorbani R. (2018). Attitudes toward spirituality and spiritual care among Iranian nurses and nursing students: a cross-sectional study. *Journal of Religion and Health*.

[21] Mystakidou K., Tsilika E., Parpa E., Smyrnioti M., Vlahos L. (2007). Assessing spirituality and religiousness in advanced cancer patients. *American Journal of Hospice and Palliative Medicine*.

[22] Fradelos E. C., Latsou D., Mitsi D. (2018). Assessment of the relation between religiosity, mental health, and psychological resilience in breast cancer patients. *Współczesna Onkologia*.

[23] Terwee C. B., Bot S. D. M., de Boer M. R. (2007). Quality criteria were proposed for measurement properties of health status questionnaires. *Journal of Clinical Epidemiology*.

[24] van der Windt R. C., Widaman K. F., Zhang S., Hong S. (1999). Sample size in factor analysis. *Psychological Methods*.

[25] Musa A. S., Pevalin D. J. (2016). Development of the Arabic spiritual care intervention‐provision scale. *Journal of Clinical Nursing*.

[26] Jahandideh S., Zare A., Kendall E., Jahandideh M. (2018). Nurses’ spiritual well-being and patients’ spiritual care in Iran. *COJ Nurse Healthcare*.

[27] Soriano G. P., Aranas F. C. T., Tejada R. S. O. (2019). *Caring Behaviors, Spiritual, and Cultural Competencies: A Holistic Approach to Nursing Care*.

[28] Papastavrou E., Karlou C., Tsangari H. (2011). Cross-cultural validation and psychometric properties of the Greek version of the Caring Behaviors Inventory: a methodological study. *Journal of Evaluation in Clinical Practice*.

[29] Fradelos E., Tzavella F., Koukia E. (2016). The translation, validation and cultural adaptation of functional assessment of chronic illness therapy-spiritual well-being 12 (facit-sp12) scale in Greek language. *Materia Socio Medica*.

[30] Fradelos E., Kapsiocha E., Tzavella F. (2019). Factors associated with psychological distress in university students and the relation to emotional intelligent and spirituality: a cross-sectional study. *Materia Socio Medica*.

[31] WHO, WHO (2020). *Process of Translation and Adaptation of Instruments*.

[32] Gliem J. A., Gliem R. R. Calculating, interpreting, and reporting cronbach’s reliability coefficient for Likert-type scales.

[33] Tzounis E., Gourgoulianis K. (2013). Spirituality and spiritual evaluation, their role in providing spiritual care. *Interscientific Health Care*.

[34] Puchalski C. M. (2001). The role of spirituality in health care. *Baylor University Medical Center Proceedings*.

[35] Albaqawi H., Alquwez N., Almazan J. (2019). Workplace spiritual climate and its influence on nurses’ provision of spiritual care in multicultural hospitals. *Religions*.

[36] Musa A. S. (2016). Spiritual care intervention and spiritual well-being. *Journal of Holistic Nursing*.

[37] Ronaldson S., Hayes L., Aggar C., Green J., Carey M. (2012). Spirituality and spiritual caring: nurses’ perspectives and practice in palliative and acute care environments. *Journal of Clinical Nursing*.

[38] Martins A., Pinto S., Caldeira S., Pimentel F. (2015). Translation and adaptation of the spirituality and spiritual care rating scale in Portuguese palliative care nurses. *Revista de Enfermagem Referência*.

[39] McSherry W., Draper P., Kendrick D. (2002). The construct validity of a rating scale designed to assess spirituality and spiritual care. *International Journal of Nursing Studies*.

[40] Delgado-Guay M. O. (2014). Spirituality and religiosity in supportive and palliative care. *Current Opinion in Supportive and Palliative Care*.

[41] Chung L. Y. F., Wong F. K. Y., Chan M. F. (2007). Relationship of nurses? spirituality to their understanding and practice of spiritual care. *Journal of Advanced Nursing*.

[42] Hoover J. (2002). The personal and professional impact of undertaking an educational module on human caring. *Journal of Advanced Nursing*.

[43] Chiang Y.-C., Lee H.-C., Chu T.-L., Han C.-Y., Hsiao Y.-C. (2016). The impact of nurses’ spiritual health on their attitudes toward spiritual care, professional commitment, and caring. *Nursing Outlook*.

